# Paratyphoid Infections

**Published:** 1902-06-21

**Authors:** 


### PARATYPHOID INFECTIONS.

The whole subject of the paratyphoid infections and of the distinctions between the various organisms by which they are produced is at present in too nebulous a condition for us to enter fully into it here. Some few words on the subject may however not be without interest. Ever since the isolation of the bacillus typhosus the points of resemblance and of difference between it and the bacillus coli communis have been a subject of constant study among bacteriologists, and in view of the number of "strains"—slight differences among bacilli which yet correspond in their general characteristics to their proper type—which have been met with both in the colon and the typhosus group, it has been impossible not to suspect that these and probably many other kinds of bacteria are but varieties of one great group of intestinal micro-organisms some of which are in their ordinary condition innocent, while others are pathogenic to man. Dr. Warren Coleman[1] and Dr. B. H. Buxton, of New York, have lately made a careful study of these so-called paratyphoid organisms. They say that since Gärtner's discovery of the B. enteritidis, increasing interest has attended the study of the intermediate members of the typhoid-colon group of bacilli. In 1893 Gilbert proposed the terms "paratyphoid" and "paracolon" to designate these intermediaries; but Durham regards these terms as inappropriate, since he believes that the intermediates are well-defined species of organisms, and suggests that they should all be classified under the group name "Gärtner and its allies." From a clinical standpoint, however, the term paratyphoid is the most clearly distinctive that can be applied to infections clinically resembling typhoid, yet caused by one of these intermediate bacilli. It must be understood that as there exist a considerable group of bacilli, so there may be a considerable range of symptoms which, according to this definition, may have to be regarded as paratyphoid; thus, according to some the B. enteritidis, and the similar organisms described in association with epidemics of meat-poisoning, certain gas-producing so-called "typhoid" bacilli, the B. psittacosis, and even the B. icteroides of Sanarelli, are all to be regarded as intermediates between the colon and the typhosus group; while in the same series there are other bacilli which, so far as their characteristics place them in the series, should stand beyond the B. typhosus, thus out-typhoiding the typhoid bacillus itself. The clinical interest of all this, however, centres itself on the fact that we may meet with cases which resemble, or indeed appear identical with typhoid, but yet appear to be caused by an intermediate bacillus, thus perhaps explaining the occasional discrepancies reported in regard to the Widal reaction. In fact, "practically all the cases of paratyphoid infection passed clinically for typhoid fever without a Widal reaction, until careful bacteriological examination revealed their true nature." The authors then go minutely into the various bacteriological differences between these organs, into all of which we need not now enter, sufficient to point out that certain anomalous cases of what appear to be typhoid fever are possibly cases of infection by organisms intermediate between that organism and the B. coli.

[1] American Journal of Medical Sciences, June, 1902.

